# Supplementary material for: Elevated CSF GAP-43 is associated with accelerated tau accumulation and spread in Alzheimer’s disease
Source: Nat Commun. 2024 Jan 3;15:202. doi: 10.1038/s41467-023-44374-w (PMC10764818; doi:10.1038/s41467-023-44374-w)
Supplement: Supplementary file 1 — Supplementary Information [file 41467_2023_44374_MOESM1_ESM.pdf]

Supplementary Information

Supplementary Table 1: Demographics of the rsfMRI control sample

Age	67.17±7.24
Sex (m/f)	16/26
MMSE	29.1±0.85
Education Years	16.5±2.5
Global tau-PET SUVR	1.07±0.90

For Continuous measures, means and standard deviations are shown, for categorical measures, absolute numbers are shown.

Supplementary Table 2: centiloid x CSF GAP-43 interaction

Connectivity matrix threshold	Tau-PET ROC ROI	Estimate	T-value	P
10%	Q1	0.0002	3.109	0.003
	Q2	0.0001	2.277	0.025
	Q3	0.00008	1.915	0.059
	Q4	0.00004	0.1394	0.167
20%	Q1	0.0002	2.911	0.005
	Q2	0.0002	2.567	0.012
	Q3	0.00009	0.1993	0.050
	Q4	0.00002	0.867	0.388
30%	Q1	0.0002	2.926	0.004
	Q2	0.002	2.661	0.009
	Q3	0.0001	1.771	0.080
	Q4	0.0000	0.952	0.344
40%	Q1	0.0002	2.926	0.004
	Q2	0.0002	2.647	0.010
	Q3	0.00008	1.791	0.077
	Q4	0.00003	0.949	0.345

Statistical indices were derived from linear regression models and display the centiloid by CSF GAP-43 interaction on tau-PET change rates in different ROIs.
