# Supplementary material for: Elevated CSF GAP-43 is associated with accelerated tau accumulation and spread in Alzheimer’s disease
Source: Nat Commun. 2024 Jan 3;15:202. doi: 10.1038/s41467-023-44374-w (PMC10764818; doi:10.1038/s41467-023-44374-w)
Supplement: Supplementary file 3 — Description of Additional Supplementary Files [file 41467_2023_44374_MOESM3_ESM.pdf]

Description of Additional Supplementary Files

File Name: Supplementary Data 1
Description: This file contains three columns that are clearly labeled, i.e. the ROI index (column 1) of the Schaefer 200 atlas, the corresponding Braak stage (column 2) and the correspondence to the temporal meta ROI (column 3).
