# Supplementary material for: Elevated CSF GAP-43 is associated with accelerated tau accumulation and spread in Alzheimer’s disease
Source: Nat Commun. 2024 Jan 3;15:202. doi: 10.1038/s41467-023-44374-w (PMC10764818; doi:10.1038/s41467-023-44374-w)
Supplement: Supplementary file 5 — Reporting Summary [file 41467_2023_44374_MOESM5_ESM.pdf]

nature portfolio

Corresponding author(s): Dr. Nicolai Franzmeier

Last updated by author(s): December 4th, 2023

Reporting Summary

Nature Portfolio wishes to improve the reproducibility of the work that we publish. This form provides structure for consistency and transparency in reporting. For further information on Nature Portfolio policies, see our Editorial Policies and the Editorial Policy Checklist.

Statistics

For all statistical analyses, confirm that the following items are present in the figure legend, table legend, main text, or Methods section.

n/a	Confirmed	
☐	☒	The exact sample size (*n*) for each experimental group/condition, given as a discrete number and unit of measurement
☐	☒	A statement on whether measurements were taken from distinct samples or whether the same sample was measured repeatedly
☐	☒	The statistical test(s) used AND whether they are one- or two-sided<br>*Only common tests should be described solely by name; describe more complex techniques in the Methods section.*
☐	☒	A description of all covariates tested
☐	☒	A description of any assumptions or corrections, such as tests of normality and adjustment for multiple comparisons
☐	☒	A full description of the statistical parameters including central tendency (e.g. means) or other basic estimates (e.g. regression coefficient) AND variation (e.g. standard deviation) or associated estimates of uncertainty (e.g. confidence intervals)
☐	☒	For null hypothesis testing, the test statistic (e.g. *F*, *t*, *r*) with confidence intervals, effect sizes, degrees of freedom and *P* value noted<br>*Give P values as exact values whenever suitable.*
☒	☐	For Bayesian analysis, information on the choice of priors and Markov chain Monte Carlo settings
☒	☐	For hierarchical and complex designs, identification of the appropriate level for tests and full reporting of outcomes
☒	☐	Estimates of effect sizes (e.g. Cohen's *d*, Pearson's *r*), indicating how they were calculated

Our web collection on statistics for biologists contains articles on many of the points above.

Software and code

Policy information about availability of computer code

Data collection	No specific software was used for data collection.
Data analysis	All analyses were computed using R statistical software 4.0.4

For manuscripts utilizing custom algorithms or software that are central to the research but not yet described in published literature, software must be made available to editors and reviewers. We strongly encourage code deposition in a community repository (e.g. GitHub). See the Nature Portfolio guidelines for submitting code & software for further information.

Data

Policy information about availability of data

All manuscripts must include a data availability statement. This statement should provide the following information, where applicable:

- Accession codes, unique identifiers, or web links for publicly available datasets
- A description of any restrictions on data availability
- For clinical datasets or third party data, please ensure that the statement adheres to our policy

All data used in this manuscript are publicly available from the ADNI database (adni.loni.usc.edu) upon registration and compliance with the data use agreement. Source data are provided with this paper.

Research involving human participants, their data, or biological material

Policy information about studies with human participants or human data. See also policy information about sex, gender (identity/presentation), and sexual orientation and race, ethnicity and racism.

Reporting on sex and gender	Sex was determined as biological sex based on clinical assessments. All findings apply for both sexes, hence we do not make any specific comments about sex-specificity of our findings.
Reporting on race, ethnicity, or other socially relevant groupings	We do not report on race, ethnicity or other groupings
Population characteristics	All population characteristics relevant for the study are shown in table 1 in the manuscript. Since these data are complex, we refer the reader to the manuscript and do not summarize descriptive statistics here.
Recruitment	The authors were not involved in patient recruitment or data acquisition. All recruitment procedures and data acquisition protocols can be found on the ADNI website (https://adni.loni.usc.edu/methods/documents/)
Ethics oversight	Ethics approval was obtained by each participating ADNI site

Note that full information on the approval of the study protocol must also be provided in the manuscript.

Field-specific reporting

Please select the one below that is the best fit for your research. If you are not sure, read the appropriate sections before making your selection.

[x] Life sciences [ ] Behavioural & social sciences [ ] Ecological, evolutionary & environmental sciences

For a reference copy of the document with all sections, see nature.com/documents/nr-reporting-summary-flat.pdf

Life sciences study design

All studies must disclose on these points even when the disclosure is negative.

Sample size	The sample size was determined based on subjects that met our inclusion criteria. We included 93 ADNI participants based on the availability of longitudinal flortaucipir tau-PET (i.e. at least one follow-up flortaucipir-PET visit), baseline florbetapir Aβ-PET and baseline CSF GAP-43 measures. No sample size calculation was performed
Data exclusions	All subjects that are formally enrolled in ADNI but that did not meet the inclusion criteria were excluded from our analyses. No other data were excluded
Replication	No replication sample was available
Randomization	No randomization was performed, as no treatment or experimentally manipulated condition was part of the study design
Blinding	Blinding was not performed, as no treatment or experimentally manipulated condition was part of the study design

Reporting for specific materials, systems and methods

We require information from authors about some types of materials, experimental systems and methods used in many studies. Here, indicate whether each material, system or method listed is relevant to your study. If you are not sure if a list item applies to your research, read the appropriate section before selecting a response.

Materials & experimental systems

n/a	Involved in the study	
[x]	[ ]	Antibodies
[x]	[ ]	Eukaryotic cell lines
[x]	[ ]	Palaeontology and archaeology
[x]	[ ]	Animals and other organisms
[ ]	[x]	Clinical data
[x]	[ ]	Dual use research of concern
[x]	[ ]	Plants

Methods

n/a	Involved in the study	
[x]	[ ]	ChIP-seq
[x]	[ ]	Flow cytometry
[ ]	[x]	MRI-based neuroimaging

Clinical data

Policy information about clinical studies

All manuscripts should comply with the ICMJEguidelines for publication of clinical research and a completedCONSORT checklist must be included with all submissions.

Clinical trial registration	https://clinicaltrials.gov/study/NCT00106899
Study protocol	All ADNI protocols can be found here: https://adni.loni.usc.edu/methods/documents/
Data collection	Imaging and Clinical data were collected at participating ADNI sites
Outcomes	NA

Plants

Seed stocks	NA
Novel plant genotypes	NA
Authentication	NA

Magnetic resonance imaging

Experimental design

Design type	NA
Design specifications	NA
Behavioral performance measures	NA

Acquisition

Imaging type(s)	T1-weighted structural MRI
Field strength	3
Sequence & imaging parameters	T1, Echo Planar Imaging
Area of acquisition	brain

Diffusion MRI ☐ Used ☒ Not used

Preprocessing

Preprocessing software	CAT12, FSL
Normalization	CAT12
Normalization template	MNI
Noise and artifact removal	For the independent sample of 42 cognitively normal controls that were used to generate the connectome template, resting-state fMRI (i.e., EPI) images were slice-time and motion corrected (i.e., realigned to the first volume) and co-registered to their respective T1-weighted images. Using rigid-transformation parameters, T1-derived grey-matter, eroded white matter and eroded cerebrospinal fluid (CSF) segments were transformed to EPI space. To denoise EPI images, we regressed out nuisance covariates (i.e., eroded white matter and eroded CSF timeseries plus six motion parameters) and applied detrending and band-pass filtering (0.01-0.08Hz) in EPI native space. To further reduce movement artifacts which may compromise connectivity assessment, 54 we performed motion scrubbing in which volumes exceeding a 0.5mm frame-wise displacement threshold were removed, as well as one prior and two subsequent volumes. All subjects had at least five minutes of resting-state fMRI remaining after scrubbing 55. Spatial smoothing was not carried out to avoid artificially enhancing functional connectivity caused by signal spilling between adjacent brain regions. Pre-processed rs-fMRI images were subsequently warped to MNI space using the CAT12-derived spatial normalization parameters.

	Subject-specific functional connectivity matrices were determined across the 200 ROIs of the Schaefer atlas as Fisher-z-transformed Pearson moment correlations between ROI-specific time-series. All individual matrices were averaged and thresholded at 30% density. The average functional connectivity was then converted to a distance-based connectivity matrix, 56 where shorter path-lengths between ROIs represent stronger connectivity, in line with our previous work 5, 20.
Volume censoring	To further reduce movement artifacts which may compromise connectivity assessment, 54 we performed motion scrubbing in which volumes exceeding a 0.5mm frame-wise displacement threshold were removed, as well as one prior and two subsequent volumes. All subjects had at least five minutes of resting-state fMRI remaining after scrubbing 55.

Statistical modeling & inference

Model type and settings	NA
Effect(s) tested	NA

Specify type of analysis: ☐ Whole brain ☒ ROI-based ☐ Both

Anatomical location(s): Schaefer atlas

Statistic type for inference<br>(See Eklund et al. 2016)	NA
Correction	NA

Models & analysis

n/a	Involved in the study
☐	☒ Functional and/or effective connectivity
☒	☐ Graph analysis
☒	☐ Multivariate modeling or predictive analysis

Functional and/or effective connectivity	Functional connectivity (Fisher z-transformed Pearson Moment correlation)
